# Spontaneous Kidney Rupture: Two Case Reports With Unusual Presentations

**DOI:** 10.7759/cureus.15332

**Published:** 2021-05-30

**Authors:** Abdullah H Yavuzsan, Ibrahim H Baloğlu, Ahmet T Albayrak, Kerem Bursali, Huseyin C Demirel

**Affiliations:** 1 Urology, Sisli Hamidiye Etfal Training and Research Hospital, University of Health Sciences, Istanbul, TUR

**Keywords:** emergency, kidney, perirenal hematoma, spontaneous rupture, emergent surgeries

## Abstract

Two patients came to the emergency department with sudden-onset abdominal pain. The first case was a 20-year-old male; a contrast-enhanced computed tomography (CT) scan revealed a 17 cm x 7 cm hematoma in the abdomen and left retroperitoneal space. Furthermore, the left kidney was not visualized by CT. With an emergent diagnostic laparotomy, the etiology of the bleeding was determined to be left atrophic-hydronephrotic kidney rupture. Thus, a left nephrectomy was performed. The second case was a 38-year-old male with a history of chronic hypertension; a CT scan revealed a 7 cm x 6 cm left perirenal hematoma. A left nephrectomy was performed due to hemodynamic instability on the second day of follow-up. A pathology specimen revealed a 1-cm renal cell carcinoma (RCC) in the kidney. In case of hemodynamic instability, spontaneous kidney rupture requires immediate surgical intervention; its causes include atrophic-hydronephrotic kidney and RCC.

## Introduction

Spontaneous kidney rupture (SKR) is a rare urological emergency that can be lethal [1]. It can present as flank or abdominal pain, hematuria, or hemorrhagic shock [2,3]. Since there is no history of trauma in SKR cases, there is usually an underlying renal disease. The most common causes of SKR are renal cell carcinoma (RCC) and angiomyolipomas [2,4]. Other causes include vasculitis, diseases causing coagulopathy, infections, and anticoagulant drug use [3]. In this paper, we present two patients with SKR, who came to our emergency department with sudden-onset abdominal pain.

## Case presentation

Case 1

**Figure 1:**
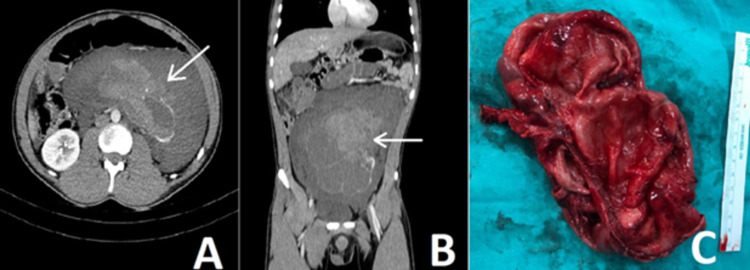
(A) Hematoma was observed at the intra-abdominal and left retroperitoneal areas in axial sections with a contrast-enhanced abdominal CT. The arrow shows a contrast extravasation consistent with active bleeding. (B) Coronal sections show hematoma that spreads to almost the entire abdomen and the arrow shows the contrast extravasation. (C) Nephrectomy material. The kidney has almost completely lost its parenchyma tissue.

A 20-year-old male patient with no history of trauma came to the emergency department with sudden onset abdominal pain. He reported a feeling of bursting and severe pain in the abdomen during weight training. The physical examination was compatible with acute abdomen findings. His intestinal sounds and rectal examination were normal. However, he had hypotension and tachycardia. The patient's urine analysis and serum creatinine level were normal. His hemoglobin level was 10.1 g/L (range 13-17.5 g/L). An abdominal contrast-enhanced computed tomography (CT) scan revealed a 17 cm x 7 cm hematoma containing active bleeding foci in the abdomen and left retroperitoneal space. Also, the left kidney was not visualized (Figures 1A, 1B). The radiologist evaluated the CT scan as a mesentery vascular injury. The patient underwent emergency laparotomy with a pre-diagnosis of mesenteric vascular injury by general surgery. No active bleeding was detected in the abdomen, except for a minimal hematoma. However, when a diffuse hematoma was observed in the left retroperitoneal area, a urology consultation was requested. The urologist drained approximately 700 cc of hematoma after entering the retroperitoneal area. Afterward, the urologist concluded that the atrophic kidney was ruptured due to severe hydronephrosis and performed a simple left nephrectomy (Figure 1C).

When the nephrectomy material was examined, fibrotic stenosis was observed at the ureteropelvic junction. In addition, the pathological analysis showed fibrosis in the ureteropelvic junction and chronic pyelonephritic changes in the renal parenchyma. The patient was stabilized after surgery and, on the third postoperative day, he was discharged.

Case 2

**Figure 2:**
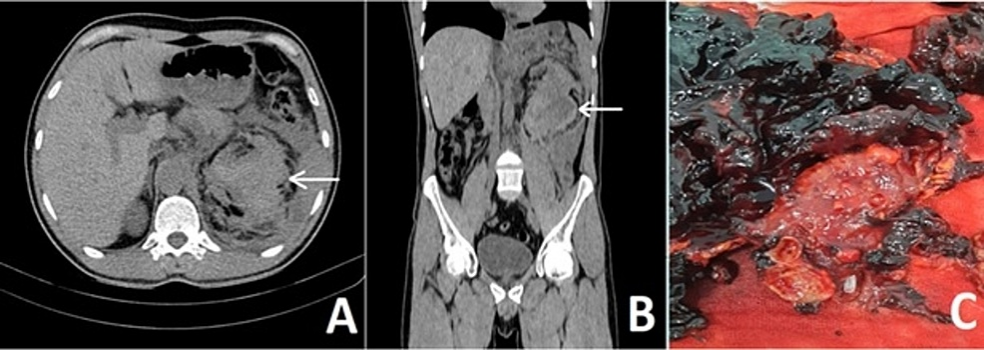
(A) Hematoma (arrow) can be seen at the posteroinferior of the left kidney in axial sections with a non-contrast abdominal CT. (B) Display of hematoma (arrow) in coronal sections. (C) Nephrectomy material. Multiple cortical cysts and solid nodular structures are seen on the kidney with the parenchymal disease.

A 38-year-old male patient came to the emergency department with sudden-onset widespread abdominal pain. Physical examination of the patient, who had no history of trauma, was compatible with acute abdomen. He had chronic renal disease and hypertension in his medical history. However, the patient, who did not use any antihypertensive medication, had not had regular nephrology follow-ups. On admission, his blood pressure was 200/140, his creatinine level was 2.55 mg/dL (range 0.7-1.2 mg/dL), and his hemoglobin level was 8.5 g/L. An abdominal non-contrast CT scan revealed a 7 cm x 6 cm left perirenal hematoma extending towards the posteroinferior of the kidney; however, no pathological condition that would cause bleeding in the kidney could be detected on the CT scan (Figures 2A, 2B). He was referred to general surgery for a consult in terms of acute abdomen; however, no intra-abdominal pathology was considered. The patient was referred to the urology clinic for follow-up as well as to the nephrology department, and appropriate antihypertensive treatment was started. Afterward, a diagnostic laparotomy with a subcostal Chevron incision was performed on the second day of follow-up due to the development of tachycardia and a decrease in hemoglobin levels despite blood transfusions. The widespread hematoma was detected in the retroperitoneum. After the hematoma was drained, a left radical nephrectomy was performed. On macroscopic examination, it was seen that the left kidney was reduced in size and compatible with the parenchymal disease. The kidney also had irregular margins, multiple solid nodular structures, and cortical cysts (Figure 2C).

In addition, pathological analysis showed total glomerular sclerosis in the left kidney consistent with end-stage renal failure. Moreover, multiple cortical adenomas and cortical cysts were found in the kidney as well as a 1-cm papillary RCC in the upper pole. After his hemoglobin level stabilized after surgery, the patient was discharged with a serum creatinine level of 2.71 mg/dL on the fifth postoperative day.

## Discussion

In this paper, we reported two SKR cases that presented with unusual symptoms. It is known that SKR can present with abdominal or flank pain, hematuria, or hypotensive shock similar to renal injuries due to trauma [5]. The first patient in our report came to the emergency room with acute abdomen-like symptoms after weightlifting exercise with no history of trauma. The second patient, who had chronic hypertension and kidney failure, also came to the emergency room with acute abdomen-like symptoms. We could not find any SKR cases in the literature, presenting with similar histories to our cases. Therefore, we think that these cases will contribute to the literature as they emphasize that the possibility of SKR should not be ignored, especially in patients who present to the emergency department with acute abdomen symptoms.

Atrophic and hydronephrotic kidneys are enlarged enough to cover almost the entire abdomen in cases of severe hydronephrosis [6]. Because of the aforementioned condition, renal perforation may develop due to increased intra-abdominal pressure. In our first case, we reported a patient who developed a kidney rupture with Valsalva maneuver during exercise. It is known that intra-abdominal pressure increases with Valsalva maneuver [7,8]. However, the mechanism of spontaneous rupture and subsequent bleeding into the subcapsular or perinephric space is unclear. The sudden rise of renal vein pressure might be the best theory as the mechanism responsible for parenchymal rupture [5]. Moreover, our patient did not know that he had an atrophic and hydronephrotic kidney. Therefore, the take-home message should be to inform the patients with severe hydronephrosis that SKR may develop due to exercise or increased intra-abdominal pressure.

Spontaneous rupture of the kidney is a rare entity that usually occurs in patients with RCC, angiomyolipoma, renal cysts, arteriovenous malformation, or vascular diseases such as periarteritis nodosa [2,9]. In the second case, the patient had RCC as well as renal cysts and renal adenoma. In addition, he had untreated hypertension. We have encountered the only case reports in the literature regarding the development of SKR in patients with nephrogenic hypertension [10]. In this report, it is seen that SKR is caused by renal hemangiomas accompanied by hypertension. In our case, we did not encounter any neoplasm, which is rich in vascular structures such as renal hemangioma or angiomyolipoma, in the kidney. This suggests that chronic hypertension may increase the risk of spontaneous bleeding in RCC, renal adenoma, or cysts, as in our case. However, there is a need for comprehensive studies on this subject involving more patients.

## Conclusions

SKR is a rare urological emergency that threatens life when appropriate treatment is not done. However, these cases may present with unusual symptoms such as sudden onset abdominal pain like in our report. It should be kept in mind that SKR can be seen in patients with an atrophic-hydronephrotic kidney or chronic renal failure where RCC, cortical adenoma and cysts may coexist.
